# Can Nutritional Screening Tools Predict the Prognosis of Critically Ill Patients with Sepsis?

**DOI:** 10.3390/medicina61101846

**Published:** 2025-10-15

**Authors:** Duygu Kayar Calili, Demet Bolukbasi, Seval Izdes

**Affiliations:** 1 Department of Anesthesiology and Reanimation-Intensive Care, Faculty of Medicine, Yıldırım Beyazıt University Ankara Bilkent City Hospital, Ankara 06800, Turkey; sevalizdes@yahoo.com; 2 Department of Intensive Care, Ankara Bilkent City Hospital, Ankara 06800, Turkey; de.emeet@hotmail.com

**Keywords:** nutritional status, sepsis, intensive care units, mortality

## Abstract

*Background and Objectives*: Although nutritional status is critical to the clinical outcomes of septic patients, studies on this topic are limited. We aim to assess the prognostic value of five nutritional screening tools (NSTs) for septic patients both at the time of admission to the intensive care unit (ICU) and five days later. *Materials and Methods*: This prospective observational study included adult septic patients in the ICU. Patients were divided into two groups: survivors and non-survivors. Clinical, laboratory characteristics, and NST values [The Controlling Nutritional Status (CONUT), Prognostic Nutritional Index (PNI), Nutritional Risk Screening (NRS-2002), Geriatric Nutritional Risk Index (GNRI), and Nutrition Risk in the Critically Ill (NUTRIC)] were recorded at admission and on Day-5, and intergroup and intragroup comparisons were performed. *Results*: A total of 126 patients were included in this study: 97 in the survival group and 29 in the non-survival group. The non-survivors had higher CONUT and NUTRIC scores and lower PNI scores. Multivariate analysis found higher Day-5 NUTRIC scores independently associated with mortality. ROC analysis identified NUTRIC > 6 as a mortality predictor. *Conclusions*: Although several markers differed significantly between survivors and non-survivors, our findings show that a high Day-5 NUTRIC score was the only factor independently associated with mortality among NSTs.

## 1. Introduction

Malnutrition has been shown to negatively affect immune function, wound healing, overall recovery, and prognosis [1,2,3]. Nutritional screening is an essential first step in guiding nutritional interventions in critically ill patients [4]. Guidelines from the European Society for Clinical Nutrition and Metabolism (ESPEN) and the American Society for Parenteral and Enteral Nutrition (ASPEN) support the use of Nutrition Risk in the Critically Ill (NUTRIC) and Nutritional Risk Screening 2002 (NRS-2002) scores for identifying nutritional risk in patients who are critically ill [5,6].

Sepsis, characterized by systemic inflammation and organ dysfunction, particularly when accompanied by underlying comorbidities, leads to high morbidity and mortality [4,7]. Metabolic changes and intense catabolic stress in critically ill patients with sepsis can exacerbate pre-existing malnutrition or trigger new nutritional deficiencies [1,8]. Various nutritional screening tools (NSTs) have been developed to identify patients’ malnutrition risk and predict outcomes in septic patients [2]. However, the complexity of the clinical picture in sepsis means it is unclear which tool is most appropriate.

The NRS-2002 is a tool that evaluates weight changes, body mass index (BMI), and dietary intake in conjunction with disease severity [6,9]. A high NRS-2002 score has been defined as a mortality-associated risk factor in critically ill patients [10]. However, research into its ability to predict mortality in critically ill septic patients is limited. The NUTRIC score, incorporating age, intensive care unit (ICU) severity scores, comorbidities, interleukin-6 (IL-6), and the time between hospital and ICU admission, has been proposed as a useful tool for predicting mortality in septic patients [8,11]. The Prognostic Nutritional Index (PNI) and The Controlling Nutritional Status (CONUT) score, which are based on laboratory markers such as total cholesterol, albumin, and lymphocyte count, have been investigated to evaluate the relationship between nutritional status and outcomes in critically ill septic patients [2,12]. The Geriatric Nutritional Risk Index (GNRI), which is calculated using serum albumin and body weight in patients aged over 65, has also been reported to correlate with mortality in sepsis [13,14].

These nutrition assessment tools differ in their components, which range from biochemical markers to anthropometric and clinical data. Their utility in septic ICU patients is an ongoing area of investigation. To the best of our knowledge, no previous study has assessed all of these NSTs concurrently and investigated changes in them over time in critically ill septic patients. We hypothesized that among critically ill septic patients, higher nutritional risk—as measured by the CONUT, NRS-2002, NUTRIC, PNI, and GNRI scores—is associated with increased mortality, and that both baseline values and their changes over the first five days of ICU stay provide prognostic insight. Thus, our primary objective was to assess the prognostic value of five NSTs—CONUT, NRS-2002, NUTRIC, PNI, and GNRI—based on their values at ICU admission and on the fifth day, in predicting mortality among critically ill septic patients. As a secondary objective, we aimed to compare laboratory and clinical parameters between survivors and non-survivors at ICU admission and on the fifth day.

## 2. Materials and Methods

### 2.1. Patients

The current study was planned as a prospective observational study. Following approval from the institutional ethics committee, we enrolled adult patients admitted to the ICU with a diagnosis of sepsis according to Sepsis-3 criteria [4]. The inclusion criteria were (1) age ≥ 18 years, (2) consent to participate, and (3) an ICU length of stay ≥ 5 days to allow collection of both admission and fifth-day laboratory and nutritional assessments.

The exclusion criteria were (1) ICU stay shorter than five days and (2) readmission to the ICU during the same hospital stay. Due to the rapid clinical and laboratory changes observed in septic patients admitted to the ICU with critical illness and undergoing a severe catabolic state, we considered that a certain duration of follow-up would be appropriate to ensure a more reliable assessment. Patients with ICU stay < 5 days were excluded to allow adequate time for observing meaningful clinical and nutritional changes, to ensure consistency in admission and fifth day comparisons, and to reduce the heterogeneity associated with early mortality or rapid discharge. The enrolled patients were categorized into two groups based on survival status: those who survived (survivor group) and those who died during ICU stay (non-survivor group). No data were missing for any of the variables included in the analysis. The mortality was analyzed based on outcomes during the patients’ ICU stay.

### 2.2. Clinical Assessment and Data Collection Instruments

The following patient characteristics were recorded: age, gender, body mass index (BMI-kg/m^2^), Acute Physiology and Chronic Health Evaluation (APACHE II) Score, Sequential Organ Failure Assessment (SOFA) Score, Charlson Comorbidity Index (CCI), comorbidities, vasopressor therapy, and length of hospital and ICU stay. Laboratory parameters [albumin (g/dL), prealbumin (g/dL), triglyceride (mg/dL), total cholesterol (TC mg/dL), procalcitonin (PCT μg/dL), C reactive protein (CRP mg/L), interleukin 6 (IL-6 pg/mL), hemoglobin (g/dL), leucocyte (×10^9^/L), and neutrophil–lymphocyte ratio (NLR)], and NST values (NRS-2002, NUTRIC, CONUT, PNI, and GNRI) were calculated twice: at ICU admission and on the fifth day of ICU stay [9,12,14,15,16].

The PNI was calculated using the formula PNI = albumin (g/L) + 0.005 × total lymphocyte count, and we used the formula GNRI = 1.489 × albumin (g/L) + [41.7 × (actual body weight/ideal body weight)] to calculate the GNRI in patients aged over 65 years.

The data recorded at admission were defined as ‘Day-0’, while data recorded on the fifth day of ICU stay were defined as ‘Day-5’. Changes between admission and Day-5 were referred to as ΔDay 0–5. Intra-group comparisons were performed to evaluate the changes in laboratory parameters and nutritional scores (ΔDay 0–5) between Day-0 and Day-5.

### 2.3. Statistical Analyses

The sample size was calculated using MedCalc version 20.218 (MedCalc Software bvba, Ostend, Belgium). Based on an area under the curve (AUC) value of 0.79, a null hypothesis value (H_0_) of 0.65, a mortality rate of 0.40, a significance level (α) of 0.05, and a power of 80% (1 − β) [17], the minimum required sample size was 117 patients. Statistical analyses were conducted using IBM version 27.0 SPSS Statistics (IBM Corp., Armonk, NY, USA) and MedCalc version 15.8 (MedCalc Software bvba, Ostend, Belgium).

Descriptive statistics (frequency, percentage, mean, standard deviation, median, and minimum–maximum values) were used to summarize patient characteristics. For categorical variables, comparisons between survivors and non-survivors were performed using the Chi-squared test, while the Independent Samples *t*-test was used for comparisons of normally distributed quantitative data. The normal distribution of continuous variables was assessed using the Kolmogorov–Smirnov test, Skewness–Kurtosis values.

For normally distributed quantitative variables, between-group comparisons (survivors and non-survivors) were conducted using the Independent Samples *t*-test, while within-group comparisons (Day-0 vs. Day-5) were assessed using the Paired Samples *t*-test. For non-normally distributed variables, between-group comparisons were performed using the Mann–Whitney U test, and within-group comparisons were conducted using the Wilcoxon signed-rank test. Spearman’s rho correlations test was used to evaluate the relationships between variables (Age and APACHE II). Binary logistic regression analysis was used to calculate odds ratios and 95% confidence intervals (CI) to identify independent predictors of ICU mortality. Receiver operating characteristic (ROC) curve analysis was performed to evaluate the discriminative ability of variables which were independently related with mortality. A *p*-value of <0.05 was considered statistically significant.

## 3. Results

This study included 126 patients, 97 of whom were discharged (the survivor group) and 29 of whom died during their ICU stay (the non-survivor group). Compared to the survivor group, the non-survivor group was younger, had higher APACHE II and SOFA scores, and received vasopressor therapy more frequently (*p* < 0.05) (Table 1). Based on the finding of a negative correlation between age and mortality, we examined the correlation between age and the APACHE II score using Spearman’s rho correlation. A negative correlation was found between age and APACHE II score (r = −0.199; *p* = 0.02).

However, no significant group differences were found in sex, comorbidities, BMI, CCI, hospital stay, or ICU length of stay (*p* > 0.05).

The non-survivor group demonstrated lower albumin levels and higher IL-6 and CRP levels on Day-5 (*p* < 0.05) (Table 2). Hemoglobin levels were lower in the non-survivor group compared to the survivor group on both Day-0 and Day-5 (*p* < 0.05). The decrease in TC values on Day-5, as well as the change from baseline (ΔDay 0–5), was greater in non-survivor patients (*p* < 0.05) (Table 2).

When the temporal changes within the groups were evaluated, a significant decrease was found in the levels of albumin, hemoglobin, and TC over time in both groups (*p* < 0.05). In the survivor group, the observed decrease in NLR, leukocyte count, IL-6, PCT, and CRP levels over time was significant (*p* < 0.05) (Table 2). However, in the non-survivor group, the changes in NLR, leukocyte count, IL-6, PCT, and CRP levels over time were not significant (*p* > 0.05).

In the non-survivor group, NUTRIC values were found to be higher, and PNI values were lower on both Day-0 and Day-5 (*p* < 0.05) (Table 3). For CONUT, a difference between the two groups was observed only on Day-5; the CONUT values of the non-survivor group were found to be higher (*p* < 0.05). There were no significant differences between the groups in terms of the NRS-2002 and GNRI values (*p* > 0.05) (Table 3).

When the temporal changes in NSTs were examined within the groups, a significant decrease in the GNRI and NUTRIC scores was found between Day-0 and Day-5 in the survivor group (*p* > 0.05) (Table 3). However, a significant decrease in GNRI and NUTRIC was not observed in the non-survivor group during this period (*p* > 0.05). The decrease in PNI values and the increase in CONUT values were significant in both groups (*p* < 0.05) (Table 3).

A regression analysis was performed on the parameters that showed significant differences between the groups. Univariate logistic regression analysis found that age, APACHE II, SOFA, vasopressor therapy, NUTRIC Day-0 and Day-5, CONUT Day-5, and PNI Day-0 and Day-5 values were associated with mortality (*p* < 0.05). However, multivariate analysis determined that age, APACHE II, vasopressor therapy, and Day-5 NUTRIC score were independently associated with mortality (*p* < 0.05) (Table 4). Subsequently, receiver operating characteristic (ROC) curve analysis was conducted for the mortality-related variables, identifying the following cut-off values: age ≤ 73 years, APACHE II score > 31, SOFA score > 6, and NUTRIC score > 6 on both Day-0 and Day-5 (*p* < 0.05) (Table 5).

## 4. Discussion

This study evaluated the relationship between various NSTs and mortality in septic ICU patients. The CONUT and NUTRIC scores were found to be higher in non-survivors, while the PNI was lower. According to the results of the multivariate regression analysis, the following factors were independently associated with mortality in ICU patients with sepsis: younger age; a higher APACHE II score; the need for vasopressor therapy; and a high NUTRIC score on Day-5. The cut-off values for predicting mortality were determined as follows: age ≤ 73 years; APACHE II score > 31; SOFA score > 6; and NUTRIC score > 6.

High APACHE II and SOFA scores indicate more severe clinical conditions and an increased risk of mortality [12,17]. Similarly, in our study, non-survivors’ APACHE II and SOFA scores were higher; however, the CCI scores did not differ between the two groups. Despite reports suggesting that frail elderly patients, often with multiple comorbidities and prolonged hospitalizations, may be more vulnerable to sepsis and mortality [18,19], our study observed higher mortality among the younger patient cohort. A higher mortality rate in younger septic patients seems paradoxical at first glance, but we observed a negative correlation between age and APACHE II score. This result leads us to conclude that younger patients in our ICU cohort had more severe illness, which consequently resulted in a higher mortality rate in this group; however, the possibility of selection bias should also be considered. Because our design excluded patients with ICU stays shorter than five days, it is possible that older patients with very early mortality or rapid recovery were underrepresented. This may have skewed the age distribution of non-survivors, resulting in a relative overrepresentation of younger, severely ill patients among deaths. This finding should also be interpreted with caution due to the sample size. We were unable to conduct subgroup analyses stratified by age categories because the limited number of deaths in our study, potential loss of power, and increased risk of over-interpretation precluded reliable results.

The use of vasopressors in hypotensive patients unresponsive to fluid resuscitation generally indicates the presence of a more serious clinical condition or severe shock [20]. In our study, the vasopressor therapy rate was higher in non-survivors, and our regression analysis showed that the need for vasopressor therapy was independently associated with mortality. Although we did not find a difference in length of stay in the ICU between the groups, previous studies have reported that length of stay in the ICU was shorter in patients who did not survive due to rapid clinical deterioration [2,11,12].

Previous studies have reported that septic patients with poor outcomes have higher levels of leukocytes, CRP, and lactate and lower levels of hemoglobin, albumin, and cholesterol [2,10,12,21]. Consistent with these findings, our study found that, compared to survivors, patients who did not survive had lower albumin, TC, and hemoglobin levels on Day-5, while the CRP and IL-6 levels were higher. Furthermore, no significant decrease in the CRP, PCT, IL-6, leukocyte count, or NLR values was observed in the non-surviving group from Day-0 to Day-5; this ongoing inflammatory response may indicate an ineffective or delayed resolution of sepsis and correlate with disease severity and mortality. In contrast, the survivors demonstrated a significant reduction in these markers over the same time period, suggesting a better immunologic response and clinical recovery. We believe that these findings emphasize the importance of dynamic rather than single-point assessments when evaluating critically ill septic patients and support the involvement of laboratory trends into clinical decision process.

Several studies have demonstrated the prognostic utility of the modified NUTRIC (mNUTRIC) score in septic and critically ill patients. In a cohort of 194 ICU patients, an mNUTRIC score > 5 was associated with higher mortality, alongside APACHE II and SOFA scores [17]. Similarly, Wełna et al. reported that an mNUTRIC score ≥ 6 was linked to vasopressor use, mechanical ventilation, renal replacement therapy, and increased mortality [11]. In another study, mNUTRIC outperformed PNI and CONUT as the strongest predictor of mortality [22]. A systematic review further identified mNUTRIC as the most reliable NST for predicting mortality and adverse outcomes in critically ill patients [23], and a meta-analysis reported that the mNUTRIC score was a powerful tool for both discriminating critically ill patients and predicting mortality [24]. In our study, we used the original NUTRIC score and determined that non-survivor patients had higher NUTRIC scores compared to survivors. Additionally, a greater decline in NUTRIC scores was observed between admission and Day-5 in the survivor group than in the non-survivor group, indicating a correlation with clinical improvement. Day-5 NUTRIC score was also independently associated with mortality. The NUTRIC score, particularly the value on Day-5, demonstrated strong predictive reliability, and a cutoff value of NUTRIC > 6 was determined for predicting mortality.

Given its strong association with mortality in our cohort, especially on Day-5, the NUTRIC score appears to be a valuable tool for identifying high-risk septic patients in the ICU. The NUTRIC score includes parameters such as APACHE II, SOFA, age, and comorbidities, which are more closely related to sepsis and disease severity; therefore, it may serve as a stronger predictor of outcomes in this patient population compared to the other NSTs. Integrating NUTRIC into routine clinical assessment may help clinicians stratify patients based on their nutritional risk and illness severity. This could prompt earlier and more targeted interventions, potentially improving outcomes in this vulnerable population. The dynamic assessment of NUTRIC over time may enhance its practical value in intensive care settings, where patient conditions can change rapidly. This approach may help in the earlier recognition of patients with increasing nutritional risk. Monitoring temporal changes in the score could provide additional information on whether the risk persists and might support more accurate prognostic assessment. In our study, a Day-5 NUTRIC score > 6 was primarily evaluated as a predictor of mortality; however, future randomized interventional studies may help to clarify whether this threshold could also serve as a trigger for nutritional interventions and whether the integration of serial NUTRIC measurements into ICU nutritional practices could potentially improve patient outcomes.

The NRS-2002 is a tool for identifying hospitalized patients at high nutritional risk, with scores ≥ 3 indicating the need for nutritional support [9]. A previous study has linked higher NRS-2002 scores to longer hospital stays and increased mortality in septic patients [10], and in a critically ill cohort, NRS-2002 has been associated with ICU length of stay, particularly when compared with other screening tools [25]. However, the applicability of the NRS-2002 in an ICU setting may be limited due to the difficulty of obtaining an accurate nutritional history or anthropometric data from critically ill patients. Based on our own clinical experience, reliably assessing recent nutritional status or weight loss in patients admitted to the ICU is often challenging, both due to the patients’ inability to respond and the unreliability of information from their relatives. Also, NRS-2002 score reflects the nutritional status of the last few weeks. Therefore, it may be insufficient for capturing acute changes in conditions such as sepsis, where a patient’s status can deteriorate rapidly. The NRS-2002 tool has limited parameters for clinical assessment, such as physiological values, organ failure, or disease severity. Consistent with these limitations, our study did not identify significant differences in NRS-2002 scores between survivors and non-survivors. We believe that while the NRS-2002 is a good tool for evaluating general nutritional risk, its ability to predict mortality may be limited in the ICU setting due to its specific dynamics.

The CONUT score is a laboratory-based tool that classifies nutritional risk from normal to severe and has been associated with worse outcomes in septic patients, including higher mortality, longer hospital stays, and greater sepsis prevalence [15,21,22]. However, the accuracy of its components may be affected by factors unrelated to nutritional status, such as inflammation or additional comorbidities, which could impact its reliability in critically ill septic patients. In our cohort, non-survivors had higher CONUT scores on Day-5; however, this association did not remain significant in multivariate analysis.

The GNRI is a tool designed for assessing nutritional status in elderly patients, with low scores linked to increased mortality and prolonged hospital stays in sepsis, as well as associations with comorbidities such as malignancy, liver disease, and renal replacement therapy [13,21,26]. Jin et al. reported that both low and high GNRI levels were associated with an increased risk of sepsis and that the prevalence of malnutrition in this population was notably high [27]. They demonstrated a U-shaped correlation, which was found in subgroup analyses to be associated with specific populations, such as obese individuals and, conversely, patients with a BMI < 30 [27]. In our study, however, GNRI did not differ between survivors and non-survivors, likely because elderly septic patients were not analyzed as a separate subgroup.

The PNI has been associated with disease severity and higher mortality in both neonatal and adult septic patients [28,29]. In a large retrospective study of 9763 septic patients, low PNI values and high CONUT scores independently predicted mortality, and PNI was also correlated with the need for mechanical ventilation [2]. In our study, we observed lower PNI values in non-surviving patients at both admission and on Day-5. However, multivariate analysis did not find PNI to be associated with mortality.

In our study, we did not find an independent association between the CONUT, GNRI, and PNI tools and mortality, which may be attributable to several factors. These tools use laboratory parameters and anthropometric data. Laboratory parameters in critically ill septic patients may be influenced by comorbidities and inflammatory status [30]. In addition, in critically ill septic patients undergoing a profound catabolic process and struggling with hemodynamic instability, rapid and significant changes can be observed in both clinical status and laboratory parameters. For example, lymphocyte levels may differ depending on whether patients are in a hyperinflammatory or immunosuppressive state. Further, since albumin is a negative acute-phase reactant with a relatively long half-life, the tools that use these values will have a reduced ability to reflect the acute process [31]. Thus, these scores may reflect acute inflammatory response rather than true nutritional status in septic patients, potentially weakening their prognostic association with mortality. Additionally, generalized edema or fluid shift into the third space in these patients may hinder the accurate determination of the actual body weight. In the early stages of critical illness, there may be uncertainties in the results of tools calculated using anthropometric measurements [32]. For this reason, we believe it is essential to consider all factors that may influence laboratory results and anthropometric measurements when evaluating the nutritional status of such patients. Moreover, the reliability and effectiveness of nutritional assessment tools that incorporate these variables should be interpreted on a patient-specific basis, particularly in critically ill populations.

Further, the exclusion of patients with an ICU stay fewer than five days may have led to a more clinically homogeneous cohort, possibly reducing the variability required to detect significant associations. In our study, the GNRI tool was applied only to patients aged 65 years and older, in accordance with its original design for geriatric populations. This constraint could have contributed to the lack of a significant association between GNRI scores and mortality in the overall cohort, as the tool does not capture nutritional risk across the entire adult ICU population. It is also possible that the absence of statistically significant findings was influenced by the limited sample size, which may have reduced the statistical power to detect subtle but clinically relevant associations.

This study has several key strengths. Firstly, the prospective design improves the accuracy of the data and reduces the bias compared to retrospective analyses. Second, the dynamic assessment of nutritional status at both admission to the ICU and on the fifth day provides valuable insights into the development of nutritional risk during the acute phase of sepsis. Furthermore, assessing five commonly used NSTs simultaneously in the same cohort allows for a thorough comparison of their predictive values. Multivariate and ROC analyses further strengthen the statistical validity of the findings, supporting the determination of clinically meaningful cut-off values. Furthermore, focusing exclusively on septic intensive care patients enhances the clinical relevance and specificity of the results.

However, this study also has some limitations. Firstly, the study was conducted at a single center, which may limit the generalizability of the results. Differences in institutional nutritional practices or sepsis management interventions may influence the results. Secondly, unmeasured confounding variables—such as actual caloric intake, protein delivery, route of nutrition and other nutritional interventions—were not evaluated and could have influenced patient outcomes. Thirdly, excluding patients who stayed in the ICU for less than five days may have introduced selection bias by omitting cases of early death or quick recovery. As a result, the findings may primarily reflect the characteristics and outcomes of more clinically stable septic patients, and caution should be exercised when generalizing these results to all patients with sepsis. Lastly, although our sample size exceeded the calculated requirement, the observed ICU mortality rate was lower than the anticipated in the power analysis. Consequently, the number of mortality events was smaller than expected, which may have reduced the statistical power of logistic regression and ROC analyses. This limitation might have caused some actual associations to go undetected in terms of statistical significance.

## 5. Conclusions

In conclusion, although several clinical and biochemical markers differed significantly between survivors and non-survivors, younger age, a higher APACHE II score, vasopressor use, and an elevated NUTRIC score were found to be independently predictive of mortality. Among the nutritional screening tools evaluated, the NUTRIC score—particularly when reassessed during ICU stay—showed the strongest prognostic value. These findings suggest that dynamic evaluation of NUTRIC may provide additional insight into patient trajectories; however, further multicenter studies with larger cohorts are needed before firm recommendations for routine clinical practice can be made.

## Figures and Tables

**Table 1:** Comparisons of Groups’ Characteristics.

		Survivors (*n* = 97)	Non-Survivors (*n* = 29)	*p*
Sex	Female	50 (51.5%)	17 (58.6%)	0.647 ^a^
	Male	47 (48.5%)	12 (41.4%)	
Age (year)		74.9 ± 13.5	66.5 ± 18.8	0.008 ^b^
BMI (kg/m^2^)		26.3 ± 5.8	25.5 ± 5.1	0.526 ^b^
APACHE II Score		26.3 ± 7.6	34.0 ± 7.1	<0.001 ^b^
SOFA Score		8.0 ± 2.8	10.1 ± 2.8	0.001 ^b^
CCI		7.5 ± 2.4	8.0 ± 2.7	0.387 ^b^
Vasopressor therapy	No	44 (45.4%)	3 (10.3%)	0.001 ^a^
	Yes	53 (54.6%)	26 (89.7%)	
Length of ICU stay (day)		23.7 ± 18.2	32.9 ± 32.5	0.154 ^b^
Length of hospital stay (day)		35.4 ± 27.3	36.7 ± 33.2	0.844 ^b^

^a^: Chi-Square Test (*n* (%)), ^b^: Independent Samples t Test (Mean ± SD), BMI: body mass index, APACHE II: Acute Physiology and Chronic Health Evaluation, SOFA: Sequential Organ Failure Assessment, CCI: Charlson comorbidity index, ICU: intensive care unit.

**Table 2:** Comparisons of Groups’ Laboratory Parameters.

		Survivors (*n* = 97)	Non-Survivors (*n* = 29)	*p* ^b^
Albumin (g/dL)	Day-0	31.0 ± 5.7	29.2 ± 6.1	0.138
	Day-5	28.1 ± 3.7	25.8 ± 3.0	0.002
	*p* ^c^	<0.001	<0.001	
	ΔDay 0–5	−2.9 ± 4.4	−3.4 ± 4.8	0.557
Prealbumin (g/dL)	Day-0	0.1 ± 0.1	0.1 ± 0.1	0.104
	Day-5	0.1 ± 0.2	0.1 ± 0.2	0.551
	*p* ^c^	0.353	0.325	
	ΔDay 0–5	0.0 ± 0.2	0.0 ± 0.2	0.709
TC (mg/dL)	Day-0	128.2 ± 46.8	123.9 ± 43.9	0.661
	Day-5	116.5 ± 45.0	91.5 ± 41.7	0.009
	*p* ^c^	0.009	<0.001	
	ΔDay 0–5	−11.7 ± 43.0	−32.4 ± 40.2	0.023
Triglyceride (mg/dL)	Day-0	138.7 ± 104.0	134.9 ± 70.6	0.851
	Day-5	134.8 ± 82.4	137.9 ± 73.8	0.853
	*p* ^c^	0.550	0.781	
	ΔDay 0–5	−4.0 ± 65.0	3.1 ± 59.2	0.602
CRP (mg/L)	Day-0	137.5 ± 109.1	155.1 ± 163.2	0.502
	Day-5	85.2 ± 73.4	138.3 ± 110.3	0.020
	*p* ^c^	<0.001	0.569	
	ΔDay 0–5	−52.3 ± 101.6	−16.8 ± 156.5	0.151
PCT (μg/dL)	Day-0	13.9 ± 30.4	9.7 ± 15.6	0.478
	Day-5	3.9 ± 10.1	7.6 ± 16.4	0.148
	*p* ^c^	<0.001	0.286	
	ΔDay 0–5	−10.0 ± 28.4	−2.1 ± 10.7	0.027
IL-6 (pg/mL)	Day-0	172.3 ± 389.3	690.4 ± 1466.4	0.070
	Day-5	74.3 ± 111.5	695.1 ± 1510.7	0.035
	*p* ^c^	0.009	0.979	
	ΔDay 0–5	−98.0 ± 364.3	4.8 ± 962.1	0.577
Hemoglobin (g/dL)	Day-0	11.0 ± 2.0	9.6 ± 1.7	0.001
	Day-5	10.1 ± 1.8	9.0 ± 1.0	<0.001
	*p* ^c^	<0.001	0.027	
	ΔDay 0–5	−0.8 ± 1.4	−0.6 ± 1.5	0.561
Leucocyte (×10^9^/L)	Day-0	14.1 ± 8.4	12.9 ± 8.3	0.488
	Day-5	10.9 ± 6.2	11.8 ± 9.5	0.649
	*p* ^c^	<0.001	0.391	
	ΔDay 0–5	−3.2 ± 7.5	−1.1 ± 7.1	0.186
NLR	Day-0	18.4 ± 16.0	15.4 ± 12.1	0.359
	Day-5	11.9 ± 13.0	14.0 ± 12.6	0.452
	*p* ^c^	<0.001	0.625	
	ΔDay 0–5	−6.4 ± 17.1	−1.4 ± 15.5	0.160

^b^: Independent Samples t Test (Mean ± SD), ^c^: Paired Samples t Test (Mean ± SD), TC: Total Cholesterol, CRP: C reactive protein, PCT: procalcitonin IL-6: Interleukin-6, NLR: neutrophil-lymphocyte ratio.

**Table 3:** Comparison of NSTs of the Groups.

		Survivors (*n* = 97)	Non-Survivors (*n* = 29)	*p* ^d^
NRS-2002	Day-0	4.0 (1.0–6.0)	4.0 (3.0–6.0)	0.888
	Day-5	4.0 (3.0–6.0)	4.0 (3.0–6.0)	0.419
	*p* ^e^	0.441	0.264	
	ΔDay 0–5	0.0 (−1.0–2.0)	0.0 (−1.0–2.0)	0.508
NUTRIC	Day-0	6.0 (1.0–10.0)	7.0 (4.0–11.0)	0.006
	Day-5	5.0 (2.0–9.0)	7.00 (4.0–10.0)	<0.001
	*p* ^e^	<0.001	0.730	
	ΔDay 0–5	−1.0 (−7.0–2.0)	0.0 (−2.0–3.0)	0.018
CONUT	Day-0	6.0 (1.0–12.0)	7.0 (0.0–12.0)	0.067
	Day-5	7.0 (1.0–12.0)	9.0 (3.0–12.0)	0.001
	*p* ^e^	<0.001	<0.001	
	ΔDay 0–5	1.0 (−4.0–6.0)	1.0 (−2.0–6.0)	0.202
PNI	Day-0	37.0 (20.0–53.0)	32.0 (20.0–46.0)	0.040
	Day-5	34.0 (22.0–43.0)	30.0 (21.0–52.0)	<0.001
	*p* ^e^	<0.001	0.011	
	ΔDay 0–5	−3.0 (−17.0–8.0)	−3.0 (−14.0–12.0)	0.843
GNRI	Day-0	93.0 (56.0–130.0)	89.0 (64.0–115.0)	0.129
	Day-5	90.0 (33.0–140.0)	83.5 (70.0–116.0)	0.126
	*p* ^e^	<0.001	0.094	
	ΔDay 0–5	−4.0 (−63.0–15.0)	−2.5 (−22.0–18.0)	0.818

^d^: Mann–Whitney U Test [Median, (Min-Max)], ^e^: Wilcoxon Test [Median, (Min–Max)], CONUT: Controlling Nutritional Status, GNRI: Geriatric nutritional risk index, PNI: Prognostic nutritional index, NUTRIC: Nutrition Risk in the Critically Ill, NRS-2002: Nutritional risk screening.

**Table 4:** Logistic Regression Analyses for Mortality.

	Univariate Logistic Regression			Multivariate Logistic Regression		
	Odds	95% CI	*p*	Odds	95% CI	*p*
Age	0.97	0.94–0.99	0.013	0.97	0.94–1.00	0.034
APACHE II Score	1.15	1.08–1.23	<0.001	1.12	1.04–1.20	0.001
SOFA Score	1.27	1.10–1.48	0.002	--	--	--
Vasopressor therapy	7.19	2.04–25.37	0.002	4.26	1.10–16.41	0.035
NRS-2002 Day-0	1.05	0.61–1.81	0.846	--	--	--
NRS-2002 Day-5	1.23	0.69–2.21	0.485	--	--	--
NUTRIC Day-0	1.43	1.11–1.85	0.006	--	--	--
NUTRIC Day-5	2.00	1.46–2.74	<0.001	2.54	1.46–4.43	0.001
CONUT Day-0	1.18	1.00–1.38	0.051	--	--	--
CONUT Day-5	1.41	1.14–1.74	0.001	--	--	--
PNI Day-0	0.92	0.86–0.99	0.024	--	--	--
PNI Day-5	0.87	0.79–0.96	0.005	--	--	--
GNRI Day-0	0.97	0.94–1.01	0.121	--	--	--
GNRI Day-5	0.98	0.95–1.01	0.225	--	--	--

APACHE II: Acute Physiology and Chronic Health Evaluation, SOFA: Sequential Organ Failure Assessment, CONUT: Controlling Nutritional Status, GNRI: Geriatric nutritional risk index, PNI: Prognostic nutritional index, NUTRIC: Nutrition Risk in the Critically Ill, NRS-2002: Nutritional risk screening.

**Table 5:** ROC analysis of mortality-related variables.

	AUC	%95 CI	Cut Off	Sensitivity	Specificity	Youden Index	+PV	−PV	*p*
Age	0.640	0.549–0.723	≤73	62.1	62.9	0.250	33.3	84.7	0.016
APACHE II Score	0.773	0.690–0.843	>31	65.5	77.3	0.428	46.3	88.2	<0.001
SOFA Score	0.704	0.616–0.782	>6	96.6	35.1	0.316	30.8	97.1	<0.001
NUTRIC Day-0	0.664	0.575–0.746	>6	65.5	63.9	0.294	35.2	86.1	0.005
NUTRIC Day-5	0.769	0.686–0.840	>6	65.5	78.4	0.439	47.5	88.4	<0.001

APACHE II: Acute Physiology and Chronic Health Evaluation, SOFA: Sequential Organ Failure Assessment, NUTRIC: Nutrition Risk in the Critically Ill.

## Data Availability

The data presented in this study are available on reasonable request from the corresponding author due to the legal and ethical restrictions. The authors do not have permission to share raw data.
